# Prospective associations between burnout symptomatology and hair cortisol

**DOI:** 10.1007/s00420-020-01528-3

**Published:** 2020-03-13

**Authors:** Johannes Wendsche, Andreas Ihle, Jürgen Wegge, Marlene Sophie Penz, Clemens Kirschbaum, Matthias Kliegel

**Affiliations:** 1 Federal Institute for Occupational Safety and Health, Fabricestrasse 8, 01099 Dresden, Germany; 2 Department of Psychology, University of Geneva, Boulevard du Pont d’Arve 40, 1211 Geneve 4, Switzerland; 3 Faculty of Psychology, TU Dresden, Technische Universität Dresden, 01062 Dresden, Germany; 4 Department of Psychotherapy and Psychosomatic Medicine, TU Dresden, University Hospital Carl Gustav Carus Dresden, Fetscherstraße 74, 01307 Dresden, Germany

**Keywords:** Burnout, Hair cortisol, Nonlinear, Prospective

## Abstract

**Objectives:**

Burnout is a stress-related, psychological syndrome due to high levels of job stressors. It has been found to be related to impairments of well-being, health, and job outcomes. Alterations of glucocorticoid secretion might be a mechanism explaining the linkage between burnout and reduced psychophysical functioning. Regarding hair cortisol as indicator this assumption, so far, has been only examined in cross-sectional studies. Therefore, we aimed to compare cross-sectional and prospective associations between different burnout symptoms and hair cortisol, additionally investigating potential nonlinear associations.

**Methods:**

The prospective study sample comprises 194 employees (95% nurses) from German geriatric care. We assessed burnout symptoms at baseline (*t*1) and 6 months later (*t*2) and collected hair samples for cortisol analyses at *t*2.

**Results:**

We found significant cross-sectional and prospective nonlinear (i.e., exponential) but not linear relationships between an aggregated measure of the burnout subscales emotional exhaustion, cynicism, and reduced efficacy and hair cortisol, even after adjusting for BMI and depressive mood. None of the single subscales of burnout was related to hair cortisol after adjusting for confounders.

**Conclusions:**

Our findings further support the assumption that accumulated burnout symptoms and hypercorticolism are positively related.

## Introduction

According to Maslach et al. (2001) burnout is a stress-related burden of employees that is defined as a psychological syndrome of three core symptoms: high levels of emotional exhaustion and cynicism (also called depersonalization), and reduced professional efficacy (also called low personal accomplishment). Burnout as job-related, long-term, and adverse strain reaction can appear in each occupational context and in each profession and develops, amongst other factors, in response to repeated and chronic job-related stressors (Leiter and Maslach 2016). International ergonomic norms now consider this concept of burnout (DIN EN ISO 10075-1 2018). In addition, in 2019, the World Health Organization (WHO) decided to include this specific definition of burnout in the 11th Revision of the International Classification of Diseases (ICD 11, QD85 Burn-out) as an occupational phenomenon but not as classified medical condition (see https://icd.who.int/en).

It has been shown that the prevalence of severe burnout symptomatology differs systematically between the countries (Eurofound 2018; Leiter and Maslach 2016; Maslach et al. 2001; Schaufeli 2018) but also across studies when considering, for instance, the well-examined profession of nurses (Adriaenssens et al. 2015; Peterson et al. 2008). Some reasons for this situation are differences in burnout definition, assessment methods (with the Maslach Burnout Inventories as most often used questionnaires), and diagnostic criteria (i.e., scale cut-offs) for burnout symptom and syndrome categorization (Bianchi et al. 2019; Leiter and Maslach 2016). In a nationwide and representative study of the German working population, Rose et al. (2016) found that 10% of male employees and 11% of female employees report severe exhaustion symptoms. However, such a definition neglects the other two core dimensions of a burnout syndrome. Putting an integrative approach that is more close to the syndromal definition outlined above and aggregating the data on all three dimensions of burnout the prevalence of a severe syndrome seems to be lower: 4% in a Finish study (Kalimo et al. 2003) and 4 to 8% in studies with Canadian hospital staff (Leiter and Maslach 2016). This is also in line with results from a representative German study showing a lifetime prevalence of 4.2% and a 12-month prevalence of 1.5% of a of diagnosed burnout syndrome by a physician or psychotherapist (Maske et al. 2016).

Systematic literature reviews revealed that burnout is associated with lower levels of well-being and health (Salvagioni et al. 2017), recovery (Wendsche and Lohmann-Haislah 2017), and more negative work outcomes such as lower job performance and higher absenteeism (Swider and Zimmermann 2010). Importantly, even at moderate levels of a burnout syndrome risks for physical and mental health problems increase (Schult et al. 2018).

With regard to the health-impairing consequences of burnout, it has been proposed that alterations in the activity of the hypothalamus pituitary-adrenal (HPA) axis might be a critical and explaining mechanism. However, findings for changes of glucocorticoid output as an indicator of chronic stress have been inconsistent (Danhof-Pont et al. 2011; Rohleder 2018). Building upon potential methodological reasons for these results, some scholars suggested that hair cortisol might be a more valid indicator for long-term glucocorticoid output (Stalder and Kirschbaum 2012). In support to this idea, Penz et al. (2018) reported cross-sectional results (*N* = 314) from a study on associations between burnout symptomatology and hair cortisol. They found significant positive associations between hair cortisol and a dichotomous score of global burnout and reduced efficacy (*β* = 0.15 for both relationships). However, no significant associations were found between hair cortisol and exhaustion (*β* = 0.03) or cynicism (*β* = 0.11). Associations were stable even after adjusting for depressive symptomatology, which might further explain such results. For instance, meta-analyses found that depression and burnout symptoms are positively correlated (e.g., Schonfeld et al. 2019; to emotional exhaustion: *r* = 0.60, to depersonalization: *r* = 0.40, to personal accomplishment: *r* = 0.33) but different and robust constructs (Koutsimani et al. 2019; burnout-depression with *r* = 0.52). Moreover, the findings of Penz et al. (2018) are in line with other cross-sectional results (*N* = 246) in a study on hair cortisol and cognitive performance (McLennan et al. 2016).

Penz et al. (2018) further suggested that nonlinear and exponential relationships might exist explaining the ‘lack of psychoendocrine covariance’ (Stalder et al. 2017, p. 270) between measures of perceived stress and hair cortisol. Specifically, only participants with extremely high values of burnout should develop elevated levels of hair cortisol. The authors found initial support for this assumption that was, however, limited by relying on a dichotomized global burnout score. Therefore, to elaborate this issue further, the first purpose of our study was to compare linear and nonlinear-exponential relationships between burnout symptomatology and hair cortisol. In contrast to the approach of Penz et al. (2018) to split the burnout scores, which might result in power problems when analyzing smaller samples, we used the continuous scores and their nonlinear exponential transformation for our analyses. This allows investigating covariation between burnout and hair cortisol in more detail.

Another caveat in the Penz et al. (2018) study is the, so far, cross-sectional analysis of data. Therefore, it stays highly speculative to assume that burnout leads to altered glucocorticoid output and not vice versa, especially with the consideration that hair cortisol is a retrospective marker. Therefore, to gain more information about possible causal directions, the second purpose of our study was to examine the time-course of associations between both variables. We used a 6-month prospective study approach and compared the prospective findings with the cross-sectional results.

## Methods

### Study design, procedure and sample

The data reported here were collected within a longitudinal study on organizational determinants of nurses’ health and work ability (ODEM study) in German geriatric care. The sampling procedure and other results are described elsewhere (Wendsche et al. 2014, 2016). With respect to data on hair cortisol, we have previously reported some cross-sectional results in a paper with the main focus on hair cortisol and cognitive performance that only used data from the second wave of the ODEM study (*t*2; McLennan et al. 2016).

At the beginning of the study (*t*1), *N* = 675 employees from facilities in German geriatric care participated in a comprehensive questionnaire survey on working conditions, job attitudes, health, and well-being. The average response rate was about 32% within organizations. This is similar to other German studies in this field (e.g., 33% in the study of Nübling et al. 2010). Participants answered the questionnaires within paid breaks at work or at home. Six months later (*t*2), a sample of *n* = 295 employees participated in the survey again (dropout of about 56%). From this sample, we were able to take a hair sample from *n* = 199 participants from which we excluded data of five participants due to outlying values (i.e., deviating more than three standard deviations from the mean). The final sample consisted of *n* = 194 persons employed in 37 care facilities. Most of the employees were nurses (95%; *n* = 11 with other professions). The mean age of employees was 40.9 years (*SD* = 11.03) and about 90% were female (*n* = 175). The average number of working hours per week was 34.08 (*SD* = 7.07) at *t*1 and 34.45 (*SD* = 6.53) at *t*2.

### Self-report measures

Participants completed a questionnaire about age (in years), sex, and height (m) and weight (kg) to calculate the Body Mass Index (BMI, kg/m^2^). Moreover, we asked for having a permanent wave (0 = ‘no’, 1 = ‘yes’), using hair coloration (0 = ‘no’, 1 = ‘yes’), and mean number of hair washes per week.

We assessed burnout symptomatology using the 16-item German Version of the Maslach Burnout Inventory-General Survey (MBI-GS; Büssing and Perrar 1992; scale range 0–6). We calculated a general burnout factor (MBI_total_: Cronbach’s *α* was 0.87 at *t*1 and 0.90 at *t*2) by the mean of all items and separate mean scores for three subscales (emotional exhaustion EE: Cronbach’s *α* was 0.83 at *t*1 and 0.88 at *t*2, cynicism Cy: Cronbach’s *α* was 0.85 at *t*1 and 0.89 at *t*2, and reduced efficacy RE which was ‘personal accomplishment’ reverse coded: Cronbach’s *α* was 0.88 at *t*1 and 0.87 at *t*2).

We used the mean of the five reverse coded items (6-point frequency scale; 1 = ‘at no time’, 6 = ‘all of the time’) from the WHO-5 well-being index (Bech 2004; Cronbach’s *α* = 0.81 at *t*1 and *α* = 0.88 at *t*2) as a screening for ‘depressive mood’ (Topp et al. 2015).

### Hair cortisol analysis

Hair strands were cut scalp-near from the posterior vertex position. We used the proximal 3 cm hair segment for analyses of hair cortisol. Based on an average hair growth rate of 1 cm/month (Wenning 2000), this represents cumulated glucocorticoid secretion over the 3-month period prior to sampling. Hair samples were stored in labeled foil packages in a dry place. The following analyses were conducted in the lab of Clemens Kirschbaum at TU Dresden. We used 7.5 mg of hair for cortisol analyses. All further wash and steroid extraction methods followed the laboratory protocol described by Stalder et al. (2012, Study II). Cortisol levels were determined using a commercially available immunoassay with chemiluminescence detection kit (CLIA, IBL-Hamburg, Germany). As raw values of hair cortisol were positively skewed, we used the log10-transformed values which showed better fit to normal distribution (Shapiro–Wilks’ *W*(194) = 0.99, *p* = 0.155).

### Data processing and statistical analyses

At first, we checked the necessity to conduct hierarchical linear modeling, because our data have a hierarchical structure with individual employees nested within organizations. The results of an ANOVA showed that employees’ hair cortisol did not significantly differ between the organizations (*F* = 0.97, *p* = 0.521). In addition, the intra-class correlation coefficient (ICC) of hair cortisol was rather low (0.027). Therefore, we conducted all analyses on the individual level.

Second, we conducted an analysis of systematic dropouts and calculated descriptive statistics (means and standard deviations) and intercorrelations for all study variables. Third, we inspected fractional polynomial prediction plots with STATA 15 to test the type of (non)linear relationship. In the next step, we examined linear and nonlinear relationships between burnout symptomatology and hair cortisol in the cross-sectional (*t*2) and prospective (*t*1, *t*2) part of data using simple and multiple regression analyses. We calculated the exponential terms of the burnout scores (e^score^) to probe nonlinear effects. For all four outcomes (MBI_total_, EE, Cy, RE) we report results of six statistical models. In Model 1–3 we tested single effects of all four burnout measures and in Model 4–6 their multiple (only subscales EE, C, RE of burnout) and combined effects (Model 2, 3, 5, and 6 with adjustment of confounders).

We set level of significance to *p* = 0.05 (two-sided) and conducted all statistical analyses with IBM SPSS Statistics 25 (SPSS Inc., IL, USA).

## Results

### Dropout analyses

We found almost no indication of a systematic dropout of participants. Employees in the final sample did not significantly differ at *t*1 in age, sex, working hours, burnout symptoms, depressive mood, and BMI from those participating only at *t*1. Moreover, we found almost no significant differences in these variables to participants at *t*2 for which the analysis of hair cortisol was not possible. For the latter analysis, however, there was a difference regarding sex distribution (*Χ*^2^ = 4.45, *p* = 0.046), indicating that there were more males (21%) in that sample for which we could not analyze hair cortisol data than in the final sample (10%). This might be because male hair strands were too short for such analyses.

### Preliminary analyses

Means and standard deviations of all variables at the baseline (*t*1) and 6 months later (*t*2) are summarized in Table 1. Regarding the potential control variables (age, sex, BMI, depression, working hours, permanent wave, coloration, hair washes) only BMI was significantly positively related to hair cortisol (*r* = 0.36 with *p* < 0.001 for cross-sectional and prospective relationships). Therefore, we decide to adjust our models for this variable. Depressive mood was not related to hair cortisol but correlated significantly positively with burnout symptomatology at both times of measurement (*r* at *t*1 and *t*2; MBI_total_: 0.51 and 0.60, EE: 0.55 and 0.58, Cy: 0.39 and 0.50, RE: 0.19 and 0.14). Moreover, there were some inconsistent but rather small-sized (all *r* < 0.22) relationships between burnout symptoms and sex, BMI, and hair washes per week. However, to keep our statistical models parsimonious and in line with the approach of Penz et al. (2018), we decided not including them as further confounders.

**Table 1:** Descriptive statistics and correlational analyses of all study variables

		*M*	SD	1	2	3	4	5	6	7	8	9	10	11	12	13	14	15	16	17	18	19
1	Age	40.91	11.03	–																		
2	Sex	0.10	0.30	− 0.16*																		
3	BMI (*t*1)	26.66	5.34	0.06	− 0.03																	
4	BMI (*t*2)	26.77	5.36	0.06	− 0.01	0.97*																
5	Depressive mood (*t*1)	3.10	0.91	− 0.08	0.07	0.17*	0.19*															
6	Depressive mood (*t*2)	3.08	0.99	− 0.07	0.12	0.10	0.12	0.54*														
7	Working hours (*t*1)	34.09	7.07	− 0.07	0.06	− 0.03	− 0.06	− 0.04	− 0.05													
8	Working hours (*t*2)	34.45	6.55	− 0.08	0.10	− 0.07	− 0.09	0.01	0.03	0.80*												
9	Permanent wave	0.01	0.07	0.13	− 0.02	− 0.05	− 0.05	0.07	− 0.08	− 0.09	− 0.10											
10	Coloration	0.79	0.41	0.02	− 0.52*	< 0.01	− 0.02	− 0.04	− 0.07	− 0.04	− 0.04	0.04										
11	Hair washes/week	3.54	1.81	− 0.28*	0.26*	0.03	0.04	0.01	0.07	0.11	0.12	− 0.10	− 0.16*									
12	MBI_total_ (*t*1)	1.27	0.77	0.03	0.12	0.13	0.15*	0.51*	0.38*	0.01	0.03	− 0.03	− 0.04	0.02								
13	MBI_total_ (*t*2)	1.35	0.85	− 0.03	0.13	0.11	0.11	0.44*	0.60*	− 0.01	0.01	− 0.03	− 0.05	0.02	0.64*							
14	EE (*t*1)	2.20	1.25	0.08	< 0.01	0.16*	0.17*	0.55*	0.37*	0.04	0.08	− 0.01	0.01	− 0.08	0.77*	0.51*						
15	EE (*t*2)	2.31	1.32	0.01	0.07	0.12	0.11	0.44*	0.58*	− 0.04	− 0.01	− 0.02	− 0.02	− 0.06	0.59*	0.86*	0.61*					
16	Cy (*t*1)	0.67	0.87	0.06	0.11	0.04	0.05	0.39*	0.35*	0.10	0.11	− 0.06	− 0.07	− 0.11	0.79*	0.66*	0.58*	0.57*				
17	Cy (*t*2)	0.88	1.11	0.03	0.16*	0.07	0.08	0.36*	0.50*	0.04	0.09	− 0.02	< 0.01	− 0.05	0.58*	0.85*	0.44*	0.73*	0.74*			
18	RE (*t*1)	1.00	1.04	− 0.05	0.17*	0.07	0.09	0.19*	0.14*	− 0.10	− 0.11	< 0.01	− 0.04	0.21*	0.67*	0.30*	0.12	0.17*	0.30*	− 20*		
19	RE (*t*2)	1.00	1.04	− 0.05	0.17*	0.07	0.09	0.19*	0.14*	− 0.10	− 0.11	< 0.01	− 0.04	0.21*	0.67*	0.30*	0.12	0.17*	0.30*	− 20*	0.99*	
20	Hair cortisol	1.02	0.38	0.08	< 0.01	0.36*	0.36*	− 0.01	− 0.05	0.05	− 0.02	− 0.07	− 0.03	0.14	0.02	− 0.04	0.04	− 0.04	0.06	0.05	− 0.05	− 0.05

The reverse coded WHO 5 scale was used as screening for depressive mood. For correlations of hair cortisol, we used the respective log10-transformed values

*N* = 194, *M* mean, *SD* standard deviation, Sex (0 = ‘female’, 1 = ‘male’), *BMI* body mass index, having a permanent wave (0 = ‘no’, 1 = ‘yes’), using hair coloration (0 = ‘no’, 1 = ‘yes’), *MBI*_*total*_ total Burnout score, *EE* emotional exhaustion, *Cy* cynicism, *RE* reduced efficacy

*p < 0.05

Penz et al. (2018) also conducted analyses with MBI_total_ as a dichotomous variable (severe symptoms with MBI_total_ > 3.5; Kalimo et al. 2003). In contrast to this sample with about 16% of participants showing severe burnout symptoms, this rate was smaller in our sample (*t*1: 1%; *t*2: 2%).

### Polynomial regression plots

**Fig. 1:**
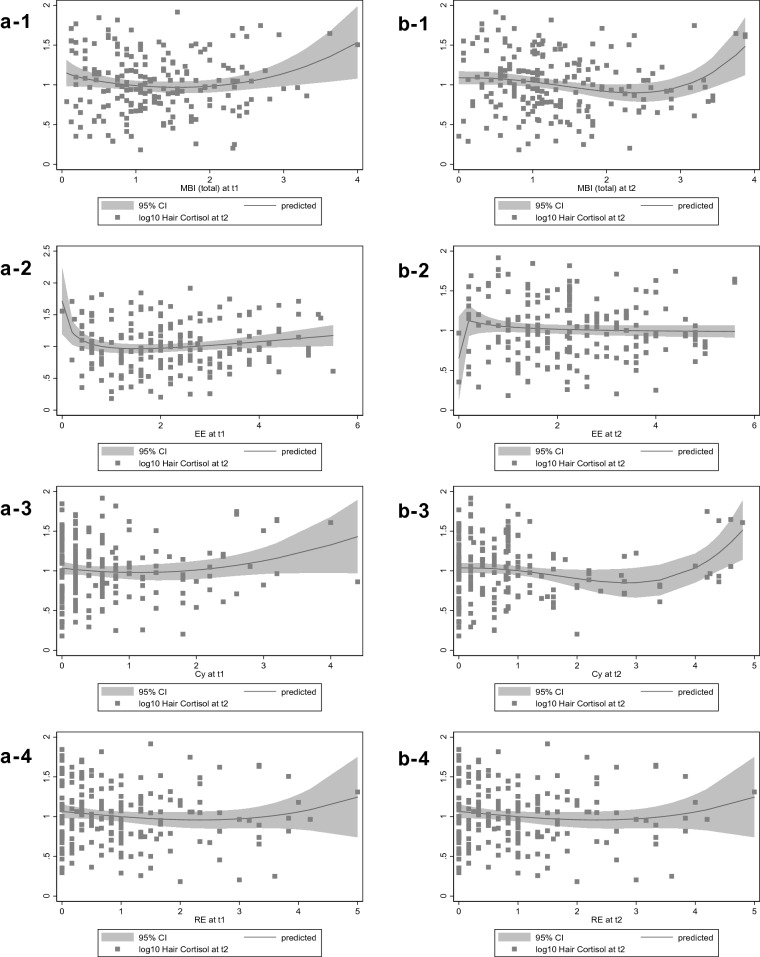
Fractional polynomial prediction plots of burnout measures and log10-transformed hair cortisol at *t*2 (1 = MBI_total_, 2 = emotional exhaustion EE, 3 = cynicism Cy, 4 = reduced professional efficacy RE) for **a** prospective (burnout measures at *t*1) and **b** cross-sectional (at *t*2, 6 months after *t*1) relationships

Figure 1 shows the fractional polynomial predictions plots of hair cortisol regressed on burnout measures including the predicted relationship and its 95% confidence interval. With emotional exhaustion as exception, we interpreted all other graphs to indicate exponential relationships. Therefore, this first analysis of graphic patterns widely supports our initial assumptions on nonlinear relationships.

### Cross-sectional analyses

The results of the cross-sectional analyses are displayed in Table 2. We found no linear relationships between hair cortisol and the different burnout measures.

**Table 2:** Results of regression analyses for predicting hair cortisol (log10-transformed) from burnout with cross-sectional data at *t*2

	Model 1		Model 2		Model 3		Model 4		Model 5		Model 6	
	*β*	*p*	*β*	*p*	*β*	*p*	*β*	*p*	*β*	*p*	*β*	*p*
*A. Linear effects model*												
MBI_total_	− 0.04	0.541	− 0.09	0.204	− 0.05	0.581						
EE	− 0.04	0.582	− 0.08	0.239	− 0.04	0.647	− 0.15	0.151	− 0.19	0.052	− 0.15	0.150
Cy	0.05	0.531	0.02	0.799	0.09	0.269	0.17	0.115	0.17	0.079	0.19	0.058
RE	− 0.05	0.478	− 0.09	0.210	− 0.07	0.281	− 0.06	0.420	− 0.09	0.200	− 0.09	0.213
*B. Nonlinear effects model*												
MBI_total_ (exp)	**0.42**	**0.004**	**0.38**	**0.005**	**0.37**	**0.006**						
EE (exp)	0.17	0.160	0.12	0.260	0.13	0.244	< 0.01	0.975	− 0.03	0.821	− 0.03	0.826
Cy (exp)	**0.29**	**0.033**	0.25	0.053	0.22	0.087	0.23	0.147	0.17	0.244	0.17	0.259
RE (exp)	0.14	0.204	0.18	0.089	0.16	0.113	0.13	0.265	0.16	0.130	0.16	0.134

*N* = 194, *β* = standardized regression weight, Model 1 = single effects analyses, Model 2 = Model 1 + BMI, Model 3 = Model 1 + BMI + Depressive Mood (WHO 5 reverse), Model 4 = multiple regression model with all three burnout symptoms (EE, Cy, RE), Model 5 = Model 4 + BMI, Model 6 = Model 4 + BMI + depressive mood (WHO 5 reverse)

*MBI*_*total*_ total burnout score, *EE* emotional exhaustion, *Cy* cynicism, *RE* reduced efficacy, *Exp* exponential term with basis e (e^Burnout score^). In the nonlinear effects model (B) exponential effects of all variables were adjusted for their corresponding linear effects. Significant effects (*p* < 0.05) are in bold face

However, in the nonlinear effects models, we found consistently that the exponential term of MBI_total_ positively related to hair cortisol and explained about 4% additional variance (Δ*R*^2^) above BMI, depressive mood and the linear term of MBI_total_. There was also some indication that in the single effects model (Model 1) the exponential term of cynicism positively related to hair cortisol (Δ*R*^2^ = 0.02). However, this effect dropped to nonsignificance when adjusting for confounders and the other burnout dimensions.

### Prospective analyses

The results of the prospective analyses are displayed in Table 3. In the linear effects models burnout symptomatology was not significantly related to levels of hair cortisol 6 months later. In contrast, we found that in the nonlinear effects models the exponential term of MBI_total_ significantly positively predicted levels of hair cortisol 6 months later (Δ*R*^2^ = 0.03) even when adjusting for BMI and depressive mood (Δ*R*^2^ = 0.02). In the linear and nonlinear effects models the subscales of burnout were not significantly related to levels of hair cortisol 6 months later.

**Table 3:** Prospective results of regression analyses for predicting (log10-transformed) hair cortisol 6 months (*t*2) after assessment of burnout (at *t*1)

	Model 1		Model 2		Model 3		Model 4		Model 5		Model 6	
	*β*	*p*	*β*	*p*	*β*	*p*	*β*	*p*	*β*	*p*	*β*	*p*
*A. Linear effects model*												
MBI_total_	0.02	0.825	− 0.03	0.656	0.01	0.902						
EE	0.04	0.582	− 0.02	0.800	0.03	0.687	< 0.01	0.978	− 0.08	0.366	− 0.03	0.724
Cy	0.06	0.410	0.05	0.495	0.09	0.224	0.08	0.382	0.12	0.164	0.13	0.139
RE	− 0.05	0.478	− 0.08	0.256	− 0.07	0.343	− 0.08	0.321	− 0.10	0.143	− 0.09	0.186
*B. Nonlinear effects model*												
MBI_total_ (exp)	**0.30**	**0.021**	**0.26**	**0.034**	**0.25**	**0.045**						
EE (exp)	0.13	0.278	0.09	0.423	0.08	0.457	0.07	0.603	0.01	0.960	< 0.01	0.995
Cy (exp)	0.11	0.351	0.10	0.355	0.10	0.338	0.07	0.542	0.05	0.651	0.06	0.573
RE (exp)	0.14	0.204	0.18	0.084	0.17	0.109	0.14	0.212	0.19	0.086	0.18	0.105

*N* = 194, *β* = standardized regression weight, Model 1 = single effects analyses, Model 2 = Model 1 + BMI, Model 3 = Model 1 + BMI + Depressive Mood (WHO 5 reverse), Model 4 = multiple regression model with all three burnout symptoms (EE, Cy, RE), Model 5 = Model 4 + BMI, Model 6 = Model 4 + BMI + depressive mood (WHO 5 reverse)

*MBI*_*total*_ total burnout score, *EE* emotional exhaustion, *Cy* cynicism, *RE* reduced efficacy, *Exp* exponential term with basis e (e^Burnout score^). In the nonlinear effects model (B) exponential effects of all variables were adjusted for their corresponding linear effects. Significant effects (*p* < 0.05) are in bold face

### Supplementary analyses

We ran a further multiple regression analysis to get more insight into the question whether the prospective or the cross-sectional linear and nonlinear global burnout scores (MBI_total_) are more predictive for hair cortisol (adjusted for BMI and depressive mood). In this model (*F*(6) = 7.14, *p* < 0.001; *R*^2^ = 0.19) only the cross-sectional linear (*β* = − 0.39, *p* = 0.010) and exponential (*β* = 0.33, *p* = 0.019) global burnout indicators were significant (prospective: *β*_linear_ = − 0.08, *p* = 0.588; *β*_nonlinear_ = 0.16, *p* = 0.217).

To keep our results in comparison to the approach of Penz et al. (2018), we used an unweighted burnout score of all items to calculate MBI_total_. However, in contrast to the subscales emotional exhaustion and cynicism, which are assessed with five items in this questionnaire, reduced personal accomplishment (reduced efficacy) is assessed with six items. Therefore, Kanthak et al. (2017) have suggested a weighted index (average of the three subscales). We repeated our analyses regressing hair cortisol on this weighted index of MBI_total_. In general, size of parameter estimates and significance of effects remained fairly unchanged. However, in the prospective analyses the nonlinear effect in Model 3 was no longer significant (*β* = 0.25, *p* = 0.051) and in the cross-sectional analyses the linear association between MBI_total_ and hair cortisol reached significance in Model 1 and 2 (*β*_Model 1_ = − 0.27, *p* = 0.031; *β*_lModel 2_ = − 0.26, *p* = 0.027) in addition to the significant exponential effect.

## Discussion

Recent models have suggested that hypercorticolism might be a mechanism that could explain how burnout symptomatology develops into impaired psychological and physical functioning (Rohleder 2018). However, data on associations between burnout and indicators of long-term glucocorticoid output, such as hair cortisol, is sparse. Moreover, the type of association and its time-course are under discussion. In this study, we wanted to shed light on both issues and compared linear and nonlinear relationships between burnout symptomatology and hair cortisol in a cross-sectional and prospective analysis of data from German employees working in geriatric care.

We were able to corroborate the cross-sectional findings of Penz et al. (2018) for nonlinear and exponential but not linear relationships between accumulated burnout symptomatology and hair cortisol. In addition, our data showed for the first time that such relationships also exist over a 6-month prospective time-course. Therefore, if burnout symptomatology accumulates employees may develop elevated levels of hair cortisol, which is in line with results on increased glucocorticoid secretion under chronic stress (Stalder et al. 2017). This is further underlined by our findings and those of Penz et al. (2018) showing that a combined burnout score, which indicates more accumulated burnout symptoms, is a stronger predictor of hair cortisol than the well-known single burnout subscales.

However, we also obtained results that are not in full agreement with findings reported by Penz et al. (2018). Our cross-sectional results indicated a weak but significant positive association between cynicism and hair cortisol, but could not find a substantial association between hair cortisol and reduced efficacy. One potential explanation for this difference is that Penz et al. (2018) reported higher mean scores and more variance in the efficacy scale in contrast to our sample. This might have contributed to these findings and future research should examine this idea further.

Whereas depressive mood correlated positively with burnout symptoms, which is in line with other study results (Koutsimani et al. 2019; Schonfeld et al. 2019), we found no significant relationships between depressive mood and hair cortisol. As results were rather stable even when adjusting for this variable (see also Penz et al. 2018), it is unlikely that depression could explain our findings.

Our study has also some limitations that have to be considered when interpreting the results reported here. First, we assessed hair cortisol only at *t*2. Hence, we were not able to adjust our analyses for baseline cortisol levels making them at risk for floor or ceiling effects. Moreover, the relatively synchronous cross-sectional and prospective results might be explained by the high stability of burnout symptoms over 6 months (*r* = 0.64 for MBI_total_). Therefore, future studies should collect data on hair cortisol repeatedly to conduct cross-lagged panel and change score analyses (see Herr et al. 2018; Penz et al. 2019 for first applications on relationships between work stressors and hair cortisol), which might help to uncover the causal patterns between variables under investigation further. A further avenue in this research might be to access physiological indicators of long-term (e.g., hair) and diurnal (e.g., blood, salvia, and urine) variations of cortisol secretion together. Although both groups of measures relate to health (Adam et al. 2017; Stalder et al. 2017), they seem differently related to subjective stress responses (van Holland et al. 2012). As timing of stressors is critical (Miller et al. 2007), collecting such data within a combined longitudinal and ‘shortitudinal’ study design (i.e., monthly/yearly and daily assessments) is highly desirable to investigate the chronification of altered burnout and cortisol levels in more detail.

Second, in contrast to the study of Penz et al. (2018) the prevalence of severe burnout symptomatology was lower in our sample. More specifically, most of our participants can by categorized according to Kalimo et al. (2003) as reporting low (baseline: 67%, *t*2: 65%) and moderate (baseline: 32%, *t*2: 33%) burnout syndrome severity. Although, we found no systematic dropout of employees with higher burnout symptoms at baseline to participate in the prospective part of this study, such persons might be still underrepresented as they are on sick leave or still have left the organization. For instance, Alarcon (2011) and Lee and Ashford (1996) showed that burnout and leaving intentions are positively correlated and in our sample the annual rate of turnover was rather high (about 17% on average; Wendsche et al. 2014). Therefore, the validity of our results is limited to the range of burnout severity in this sample and future studies should examine if our assumptions and findings also hold with samples that incorporate a higher proportion of working employees with serious burnout. Third, in this sample, but also in the study of Penz et al. (2018), females were overrepresented what limits the generalization of results to both sexes. While in our case a dominance of female employees is typical for work in the nursing sector (see also Nübling et al. 2010), we found some evidence that male workers might be systematically underrepresented, because an appropriate hair sampling is less available for them. Results of meta-analyses suggest that females report burnout symptoms more often (Purvanova and Muros 2010) but show lower levels of hair cortisol than males (Stalder et al. 2017). In our sample, we found that sex is unrelated to the average burnout score and levels of hair cortisol, which corresponds to the meta-analytically small sized effects. A perspective for future studies might be to intensify sampling to increase the proportion of males, especially those with longer hair, and to repeat our analyses stratified for sex.

Finally, work characteristics such as high job demands and low job resources (Alarcon 2011; Lee and Ashford 1996) that have been found as drivers of burnout development were widely not considered in our analyses. In our sample, working hours as indicator of quantitative demands did not relate to burnout symptomatology and hair cortisol. However, some current studies showed that task-related and organizational job demands such as client aggression (Kind et al. 2018), surface acting (Qi et al. 2017) and effort-reward-imbalance (Herr et al. 2018; van der Meij et al. 2018) are related to higher levels of hair cortisol. However, since such job conditions are considered as antecedents of burnout it is less likely that they might fully explain the relationships to hair cortisol reported here. Nevertheless, future studies should consider job demands and burnout in combination when predicting hair cortisol.

In conclusion, our results support to the assumption that burnout and long-term hypercorticolism are positively related, in particular, if burnout symptoms accumulate. In this respect, it is important to consider nonlinear modeling when examining associations between burnout and hair cortisol.
