# Correction to: Prospective associations between burnout symptomatology and hair cortisol

**DOI:** 10.1007/s00420-021-01758-z

**Published:** 2021-10-12

**Authors:** Johannes Wendsche, Andreas Ihle, Jürgen Wegge, Marlene Sophie Penz, Clemens Kirschbaum, Matthias Kliegel

**Affiliations:** 1 Federal Institute for Occupational Safety and Health, Fabricestrasse 8, 01099 Dresden, Germany; 2 Department of Psychology, University of Geneva, Boulevard du Pont d’Arve 40, 1211 Geneve 4, Switzerland; 3 Faculty of Psychology, TU Dresden, Technische Universität Dresden, 01062 Dresden, Germany; 4 Department of Psychotherapy and Psychosomatic Medicine, TU Dresden, University Hospital Carl Gustav Carus Dresden, Fetscherstraße 74, 01307 Dresden, Germany

## Correction to: International Archives of Occupational and Environmental Health (2020) 93:779–788 10.1007/s00420-020-01528-3

The article Prospective associations between burnout symptomatology and hair cortisol, written by Johannes Wendsche · Andreas Ihle· Jürgen Wegge · Marlene Sophie Penz · Clemens Kirschbaum · Matthias Kliegel was originally published electronically on the publisher’s internet portal (currently SpringerLink) on 13 March 2020 without open access.

With the author(s)’ decision to opt for Open Choice the copyright of the article changed on September 15th 2021 © The Author(s) 2021 and the article is forthwith distributed under the terms of the Creative Commons Attribution 4.0 International License (http://creativecommons.org/licenses/by/4.0/), which permits use, duplication, adaptation, distribution and reproduction in any medium or format, as long as you give appropriate credit to the original author(s) and the source, provide a link to the Creative Commons license and indicate if changes were made.

Open Access This article is distributed under the terms of the Creative Commons Attribution 4.0 International License (http://creativecommons.org/licenses/by/4.0/), which permits unrestricted use, distribution, and reproduction in any medium, provided you give appropriate credit to the original author(s) and the source, provide a link to the Creative Commons license, and indicate if changes were made.

The original article was corrected.

